# Development of an optimised method for the analysis of human blood plasma samples by atmospheric solids analysis probe mass spectrometry

**DOI:** 10.1016/j.ijms.2024.117386

**Published:** 2025-02

**Authors:** Annabel S.J. Eardley-Brunt, Anna Jones, Thomas Mills, Liwen Song, Rafail Kotronias, Pierfrancesco Lapolla, Oxford Acute Myocardial Infarction (OxAMI) Study, Oxford Abdominal Aortic Aneurysm (OxAAA) Study, Ashok Handa, Regent Lee, Keith Channon, Giovanni Luigi de Maria, Claire Vallance

**Affiliations:** a Department of Chemistry, University of Oxford, Chemistry Research Laboratory, 12 Mansfield Rd, Oxford, OX1 3TA, UK; b NIHR Oxford Biomedical Research Centre, John Radcliffe Hospital, Oxford University Hospitals, Oxford, UK; c Division of Cardiovascular Medicine, Radcliffe Department of Medicine, University of Oxford, Oxford, UK; d Nuffield Department of Surgical Sciences, University of Oxford, Oxford, UK

**Keywords:** Plasma, Metabolite profile, Atmospheric solids analysis probe mass spectrometry, Protocol optimisation

## Abstract

Analysis of small-molecule metabolites in plasma has the potential for development as a clinical diagnostic and prognostic tool. Atmospheric solids analysis probe mass spectrometry (ASAP-MS) is capable of performing rapid metabolite and small molecule fingerprinting, and has the potential for use in a clinical setting. Combining ASAP-MS data with a predictive model could provide clinicians with a rapid patient risk metric, anticipating disease progression and response to treatment, and thereby aiding in treatment decisions. In order to develop predictive models, experimental errors and uncertainties must be minimised, requiring a robust experimental protocol. In the present study we have performed ASAP-MS measurements on plasma samples from patients recruited for two prospective clinical studies: the Oxford Acute Myocardial Infarction (OxAMI) study; and the Oxford Abdominal Aortic Aneurysm (OxAAA) study. Through a carefully designed series of measurements, we have optimised the method of sample introduction, together with a number of key instrument and data acquisition parameters. Following the optimisation process, we are consistently able to record high quality mass spectra for plasma samples. Typical coefficients of variation for individual mass peaks are in the range from 20%–50%, overlapping with those obtained using more sophisticated LC-MS approaches. The measurement protocol optimises mass spectral quality and reproducibility, while retaining the simplicity of measurement required for use in a clinical setting. While the protocol was developed using plasma samples from two specific patient cohorts, the method can be generalised to any plasma measurements.

## Highlights

• ASAP-MS can perform rapid metabolite and lipid fingerprinting in clinical samples.
• Combining the data with a predictive model offers a route to new patient risk metrics.
• Generation of high-quality data sets requires a robust experimental protocol.
• We have developed such a protocol for measurements on human blood plasma.

## Graphical abstract

**Figure:**
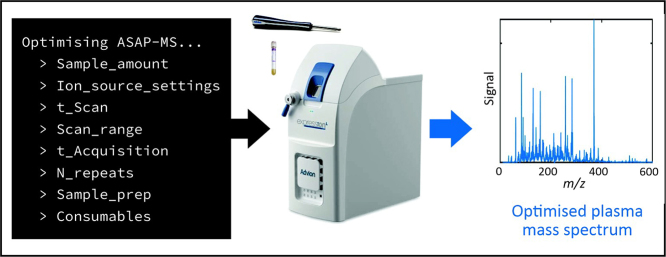

## 1 Introduction

The ability to perform untargeted metabolite profiling has gained popularity as a method of disease fingerprinting that avoids the need for slow and costly metabolomics studies on individual metabolites [1], [2], [3], [4], [5]. Taking a pattern-recognition approach to the analysis of biomarkers allows identification of metabolite fingerprints corresponding to disease phenotypes. Machine learning is well suited to solving this type of pattern recognition problem, enabling the development of predictive disease models [6], [7]. As well as providing a useful diagnostic tool in a timely manner, this type of approach can guide further research into disease pathways by identifying likely candidate biomarkers. Untargeted approaches are resource efficient and not confined to known molecular space. This is an attractive feature given that in a typical metabolomics experiment very few metabolite signals (often < 2%) can be assigned with confidence to a specific molecule; this is much lower than typical assignment rates in human proteomics and genomics experiments [8], [9], [10], [11].

A range of ambient ionisation mass spectrometry techniques have been developed which allow the sample to be introduced to the instrument unmodified, without contamination from any solvents or standards, and under environmental conditions similar to the natural state of the matrix [12], [13]. Atmospheric solids analysis probe mass spectrometry (ASAP-MS) is a bench-top rapid analytical method for conducting small molecule profiling of solid and liquid-phase samples under atmospheric conditions [14], and appears to be a promising candidate for measurements on clinical samples [15], [16]. ASAP-MS instruments are intuitive to use, and require very little sample preparation, making them potentially suited for use in a clinical setting. In the case of blood plasma, a mass spectrometric fingerprint of the metabolite and lipid content is generated within just a few seconds. Interpretation or comparison of these fingerprints using standard multivariate statistical tools or machine learning methods offers a route to untargeted metabolite profiling, with potential applications in disease diagnosis, as a prognostic tool, or to assist in making treatment decisions.

Despite these very welcome technological developments, conducting rapid measurements on plasma samples presents a number of challenges. Key amongst these is distinguishing clinically relevant metabolite changes from natural patient-to-patient variability associated with unrelated factors. Ensuring that the experimental measurements are robust and reproducible in order that experimental uncertainties are minimised is therefore essential, so that the true differences between the mass spectra of different patient groups are, correspondingly, maximised [17], [18]. Any algorithms or models applied to the data will then have the best chance of identifying the correlations of interest.

Of equal importance to the measurements themselves is ensuring the robust design of the sample collection procedures and consistent handling of the clinical samples throughout their entire life cycle. Achieving overall success requires careful design of the clinical research study protocol and clinical sample curation, alongside optimisation of all measurements yielding raw data for analysis. For the purposes of the present work, two prospective clinical research studies have been identified with stringent blood plasma curation protocols [19], [20], [21], [22] from which to source platelet/cell free plasma samples [23]. In the following, we discuss the optimisation of an ASAP-MS method for the analysis of human blood plasma samples from arterial and venous sources.

## 2 Materials and methods

Human blood plasma samples used in this study were collected as part of the OxAMI [19], [20], [21] and OxAAA [22] clinical studies at the John Radcliffe Hospital in Oxford, UK.

**OxAMI samples:** Details regarding the OxAMI study cohort have been published previously [19], [20], [21]. All participants gave written informed consent (Ethics Ref: 10/H0408/24). Coronary aspirate blood samples were obtained from OxAMI patients taken during primary percutaneous coronary intervention to restore coronary artery blood flow after a STEMI.

**OxAAA samples:** Details regarding the OxAAA study cohort and recruitment process have been published previously [22]. This single centre prospective study (Ethics Ref: 13/SC/0250) recruited AAA patients in the National Health Service setting. Each participant gave written informed consent. At the study assessment, a fasting venous blood sample was collected.

**Plasma preparation protocol:** The same plasma preparation protocol was employed in both research studies, ensuring consistent quality of samples. Blood samples were collected in EDTA tubes. Platelet-poor plasma (PPP) was prepared immediately after blood collection at room temperature using two-stage centrifugation (1st stage: 1300 g × 12 min; 2nd stage: 2500 g × 15 min) as previously described by Lee et al. [23]. Plasma was snap-frozen in dry ice and stored in a freezer at −80°C until measurements were made.

**ASAP-MS measurement:** Mass spectrometry analysis of plasma samples was conducted using an Advion express**ion**® compact mass spectrometer (CMS) ASAP-MS instrument. Data acquisition was controlled using Advion Mass Express software (version 6.9.38.1) and Advion Data Express data manipulation software (version 6.9.38.1). Instrument calibration was conducted daily in positive ion mode using Advion APCI Calibration/tune standard mix diluted with a 50:50 solution of methanol:water. Since we will be describing the optimisation of a number of instrument settings, we provide a brief description of the instrument operation in the following.

**Fig. 1:**
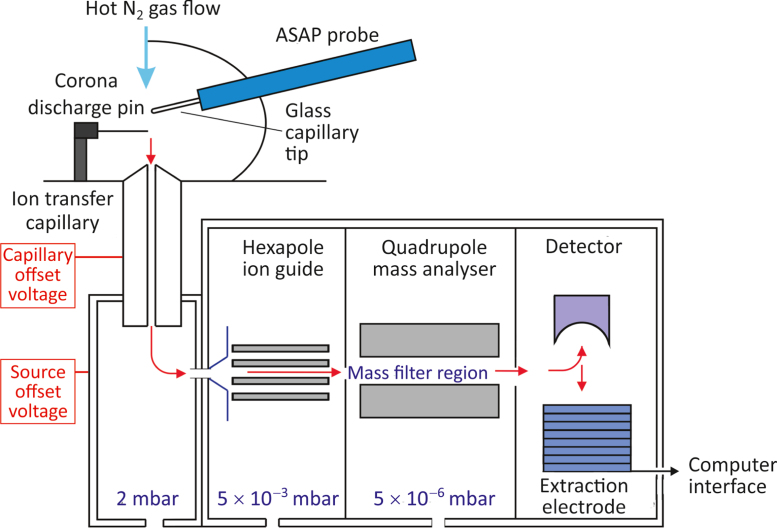
Schematic of the ASAP-MS instrument. See text for description.

Adapted from the *Advion CMS User Manual*[24]

A schematic diagram of the spectrometer is shown in Fig. 1. To make a measurement, a glass capillary tip mounted into the ASAP probe is exposed to the solid or liquid sample and inserted into the ASAP-MS ion source housing. The capillary is heated and the sample is vapourised by a flow of hot N_2_ nebulising gas. The resulting vapour is ionised via atmospheric pressure chemical ionisation initiated by a corona discharge. Ions are drawn into the ion inlet skimmer by an electric field and pressure differential at the atmospheric pressure interface. Collision-induced dissociation can occur at this interface, the extent of which may be controlled by altering the inlet potential (‘Capillary offset voltage’) and therefore the collision energy with background gas. The ions entering the inlet skimmer are collimated within a hexapole ion guide and directed to the quadrupole mass filter. The quadrupole voltages are scanned repeatedly to record a series of time-resolved mass spectra over a user selected range of mass-to-charge ratios, m/z [24], [25], [26]. The instrument can record spectra in positive or negative ion mode, or by using the ‘ion source switching mode’ can record both within a single measurement. Early tests showed that very little signal was observed for plasma samples in negative ion mode, so all data reported here employed positive ion mode only.

The glass capillaries used for sample loading (Advion ASAP S01 short) were sterilised by heating in an oven for 30 min at 250 °C and were then stored in a dessicator. After mounting a capillary into the probe tip and before recording spectra for a sample, a background spectrum of the clean glass capillary was recorded by inserting the probe into the probe slot within the ion source housing of the mass spectrometer for 30 s. The probe was removed from the source housing after the background interval, cleaned and cooled with methanol, and dried on a lens tissue.

Frozen 1 mL aliquots of plasma were thawed and vortexed immediately prior to measurement. For each measurement, a glass capillary was mounted into the tip of the ASAP probe, a small amount of sample was transferred to the capillary tip (see Section 3.1 for optimisation of this process), and the probe was inserted into mass spectrometer for the chosen acquisition time, during which a time series of mass spectra was recorded. The probe was then removed and cleaned with methanol and lens tissue before the next repeat measurement of the sample. Based on an evaluation of repeat background measurements made during a test series of sample measurements, each capillary was used for five repeat measurements and then replaced with a clean capillary.

The mass spectrometer is generally run continuously while recording background and sample spectra for an individual sample, with the spectrometer outputting the time-dependent mass spectra as a 2D array of intensities for each m/z ratio and time point in the acquisition series. Data sets were analysed using MATLAB R2022a and Python 3.7 software written in-house. The first step of data processing is to extract the individual measurements (background measurements and individual sample measurement repeats) from the large data file, corresponding to the measurement times when the probe was mounted in the ion source housing. This is an automated process based on analysis of the total ion signal as a function of time. Following background subtraction from each sample repeat, various additional data processing steps can be carried out as desired. These include averaging the data over the acquisition time, data binning (e.g. to unit m/z to reduce file sizes), and averaging over repeat measurements.

The various optimisation steps are presented in the following section.

## 3 Results and discussion

Determining the optimal approach to recording ASAP mass spectra for plasma samples requires optimisation of a variety of instrument settings, as well as consideration of a number of other factors. As noted earlier, the primary aims are to collect high-quality and reproducible mass spectra, with the secondary objective of developing a protocol that is sufficiently short and simple to be suitable for adaptation to a clinical settings. The parameters optimised in the present study are:

• **Amount of sample**: the amount of sample introduced to the mass spectrometer on each measurement;
• **Ion source settings**: The temperature and voltage settings employed within the ion source region;
• **Scan time**: The time period over which the quadrupole voltages scan through the m/z range in an individual scan;
• **Scan range**: The m/z range scanned by the quadrupole;
• **Acquisition time**: The time period for which the ASAP probe is inserted into the mass spectrometer for each sample measurement;
• **Number of repeat measurements**: The number of repeat measurements to take for each sample. These are averaged to generate the final mass spectrum for the sample for use in later data analysis.

Before working through the optimisation of each of these parameters in turn, there are few other important experimental considerations worth noting.

**Fig. 2:**
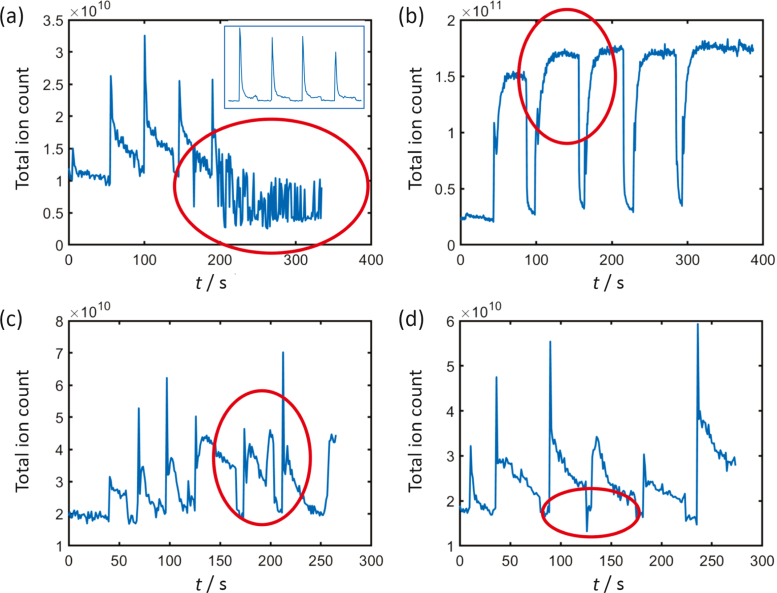
Examples of non-optimal chromatograms for OxAAA plasma samples arising as a result of various measurement errors. The resulting problems are highlighted by red circles: (a) Highly variable background and noise arising when the ion source needs to be cleaned; (b) Signal saturation due to introduction of too much sample (no sharp peak observed, and peak intensity remains high as long as the probe remains in the spectrometer); (c) Variable peak profiles and intensities due to inconsistent sampling and introduction of too much sample to the spectrometer; (d) Inconsistent acquisition times and insufficient time between measurements for the total ion count to return to background levels. The inset to (a) shows the peak shapes expected for a set of repeat measurements performed under optimised conditions. This is a subset of the data set shown in Fig. 7(a).

Even with an optimised protocol, over time residual sample will build up on surfaces within the ion source. This can lead to considerable variability in subsequent measurements (see example in Fig. 2(a)), which is resolved by cleaning the source according to the manufacturer’s instructions. The frequency with which the ion source should be cleaned is dependent on both the sample type and the frequency of use. As an example, in our own laboratory we clean the source daily, after around 150 individual measurements. Fatty samples leave more residue and may necessitate more frequent cleaning. A good ‘rule of thumb’ is that cleaning is required when the background signal is observed to rise by 10%, or when artefacts begin to appear in the chromatograms (plots of total ion signal vs acquisition time).

During method development, any consumables and solvents used should be assessed for the potential to introduce unwanted additional peaks into the mass spectra. Sample vials, solvents, ASAP capillaries, and cleaning wipes should all be considered and tested. The selection of consumables and cleaning regimen should be developed so that the minimum possible number of additional peaks are introduced. Baking the glass capillaries used in the ASAP probe for 30 mins at 250 °C was found to be a suitable method of cleaning prior to their use in sample delivery. It was also found that the same capillary can be used for sequential repeat measurements of the same sample if it is cleaned sufficiently well in between measurements. A suitable method of cleaning the capillary is to wipe the tip with methanol and a lens tissue. The capillary can then be further cleaned if necessary by inserting it into a heat source such as a butane flame or the ASAP-MS source housing for a few seconds. Once the capillary has cooled, it can then be used to conduct the next repeat measurement on the sample.

Finally, some sources of variation between measurements must be dealt with during the data processing and analysis phase. Key amongst these is correction for the amount of sample introduced into the spectrometer on each measurement via an appropriate normalisation process. Spiking the sample with an internal standard is one approach, but adds complexity to the sample preparation protocol, and is generally not desirable. Various different data scaling and normalisation methods are available, and in general it is worth trying several of these to find the most appropriate approach for a given study.

We now consider each of the parameters listed above in turn.

### 3.1 Amount of sample

While ASAP-MS has the advantages of allowing very quick and easy measurements, with minimal sample preparation required, the manual transfer of sample to the capillary tip of the probe has the consequence that the amount of sample introduced into the mass spectrometer varies on each measurement. Optimising the amount of sample transferred to the capillary tip is extremely important in order to obtain high quality mass spectra. The most common error is to transfer too much sample to the probe tip, so one should generally err on the side of transferring too little rather than too much sample. In the present work on plasma samples, the thawed samples were first vortex mixed to ensure homogeneity, and then rather than dipping the probe into the plasma, sample was transferred to the probe tip by wiping it against the inside surface of the sample container approximately 5 mm above the sample surface. The thin layer of residual sample coated onto the surface after vortexing allows a small enough sample to be introduced into the spectrometer, whereas sampling from the bulk introduces far too much sample, as discussed further in the following.

During a measurement, one generally monitors the total ion current recorded by the mass spectrometer as a function of time, known as a chromatogram. An example of the peak shapes expected for a set of well recorded chromatograms over four repeat measurements of the same sample, recorded using the sampling technique just described, is shown in the inset to Fig. 2(a). These peaks are a subset of the data set that will be shown in full and discussed in detail later (see Fig. 7(a) and associated discussion). The ion signal is strongly time dependent following insertion of the probe on each repeat. The total ion signal initially rises very rapidly as the most volatile molecules are vapourised, then falls off more slowly over a few tens of seconds until all of the sample has been desorbed. It is worth noting that the intensities of individual m/z peaks will show different time dependences, depending on factors such as the molecular enthalpy of desorption, vapourisation, or sublimation, the concentration of the molecule at the surface, and any interactions with other molecules in the sample. In contrast to the expected behaviour just described, Fig. 2(b) shows a chromatogram recorded under conditions in which too much sample was introduced into the spectrometer. On insertion of the probe, the ion current rises to a high value and does not degrade with time, a clear sign of instrument saturation. Not only does this lead to highly unreliable m/z peak intensities, it will also lead to rapid contamination of the ion source with residual sample and a need for frequent cleaning. Introduction of too much sample can also lead to highly variable peak profiles and intensities, including ‘double peaks’ in the chromatogram; an example of these effects is shown in Fig. 2(c). While the peaks in the chromatogram will vary slightly in intensity even with the best sampling technique, with some practice it should be possible to obtain repeat chromatogram peaks that are similar in shape and intensity.

### 3.2 Ion source settings

The composition of the ion cloud entering the mass analyser is strongly dependent on both the temperature of the N_2_ gas flow that desorbs sample from the probe tip, and on the potential through which the ions are accelerated into the inlet of the hexapole ion guide. At low temperatures only the most volatile molecules are desorbed, while at higher temperatures a wider range of molecules enter the gas phase. The Advion ASAP-MS instrument has pre-set temperature settings of ‘low’ (LT, 135 °C), ‘medium’ (MT, 200 °C), and ‘high’ (HT, 250 °C).

Increasing the acceleration potential of the ions as they enter the ion transfer capillary increases the energy of the collisions they undergo with the background gas. Increasing the collision energy increases the amount of collisional dissociation, in which larger ions break apart into smaller fragments. This leads to a reduction in the number of parent ions and an increase in the number of daughter or fragment ions observed in the mass spectra. The Advion ASAP-MS instrument has three pre-set acceleration potentials, accessed via the ‘high fragmentation’, ‘medium fragmentation’, and ‘low fragmentation’ ion source settings. When performing metabolic profiling, it is generally advantageous to minimise the extent of fragmentation in order to maximise the chances of matching mass spectrometric biomarkers to known molecular species within the sample. In addition, many metabolites dissociate to give one or more common fragments, which can make interpretation more challenging and obscure correlations within the data.

**Fig. 3:**
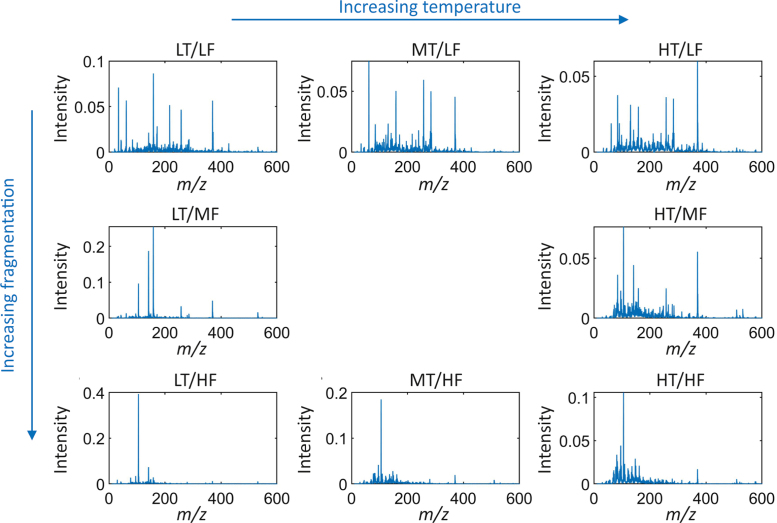
Mass spectra recorded for a human plasma sample using the eight different combinations of ion source settings listed in Table 1. The spectra were recorded with a scan time of 500 ms and an acquisition time 20 s. The spectra shown are an average over five repeats, each averaged over the 40 time-resolved spectra recorded during each acquisition time. ‘LT’, ‘MT’, and ‘HT’ refer to low, medium, and high temperature, respectively, while ‘LF’, ‘MF’, and ‘HF’ refer to low, medium, and high fragmentation.

Given the above considerations, we predicted that the ‘high temperature, low fragmentation (HT/LF)’ setting would be optimum for plasma metabolic profiling: the high temperature would yield the largest variety of gas-phase molecules, while the low fragmentation setting would minimise dissociation so that most species are detected as intact parent ions. To test this, data were recorded for all eight available combinations of ion source settings, as listed in Table 1. The results are shown in Fig. 3. As the fragmentation setting is increased from ‘low’ to ‘high’, a greater number of lower mass ions are observed, as expected. As the temperature is increased, the total ion signal is seen to increase, and a higher number of higher-mass ions, formed from less volatile molecules within the sample, are observed. As predicted, the ion source settings of HT/LF was determined to be optimal, as this maximised both the intensity and the variety of intact molecules detected from the samples. These settings were used in all subsequent measurements.

**Table 1:** Ion source settings. The capillary and source offset voltages are applied to the ion transfer capillary and source region respectively (see Fig. 1). The source voltage is scanned from the initial value at m/z=0 across the range defined by the source voltage span parameter as the mass is scanned.

Ion source setting	Capillary Temperature/ °C	Capillary offset voltage/V	Source voltage offset/V	Source voltage span/V	Gas Temperature/ °C	Corona discharge current/μA
LT/LF	135	120	20	0	250	5
LT/MF	135	160	30	5	250	5
LT/HF	135	180	40	20	250	5
MT/LF	200	120	20	0	350	5
MT/HF	200	180	40	20	350	5
HT/LF	250	120	20	0	400	5
HT/MF	250	160	30	5	400	5
HT/HF	250	180	40	20	400	5

### 3.3 Scan time

The scan time chosen to scan across the m/z range of interest determines the number of mass spectra recorded while the sample is being desorbed. A slow scan results in detection of many ions per m/z step, and therefore a high signal-to-noise ratio, but risks missing some of the most volatile species if the scan is longer than the time period over which they desorb from the sample surface. In contrast, a fast scan yields high time resolution, but lower signal-to-noise. As noted above, each chemical species within the sample has its own characteristic desorption curve, with different species yielding a peak signal intensity at different times after insertion of the probe into the ion source. In general, the signals associated with lighter, more volatile molecules tend to peak at earlier times, while those from heavier and less volatile molecules peak at later times. The optimum scan time to choose is therefore sample dependent, and is a key parameter to be investigated in any ASAP-MS study.

To determine the optimum scan time, measurements on human plasma were performed using scan times ranging from 200 ms to 1000 ms at 100 ms intervals. For each scan speed, five repeat measurements were recorded, with the probe inserted into the mass spectrometer for 20 s on each measurement. Scans were performed over the full 10–1200 m/z mass range of the instrument. The corresponding scan speeds (in seconds per unit m/z) are shown in Table 2.

**Table 2:** Instrument scan time for a scan range of 10–1200 *m/z*.

Scan time/ms	Scan speed/m/z s^−1^
200	5950
300	3967
400	2975
500	2380
600	1983
700	1700
800	1488
900	1322
1000	1190

**Fig. 4:**
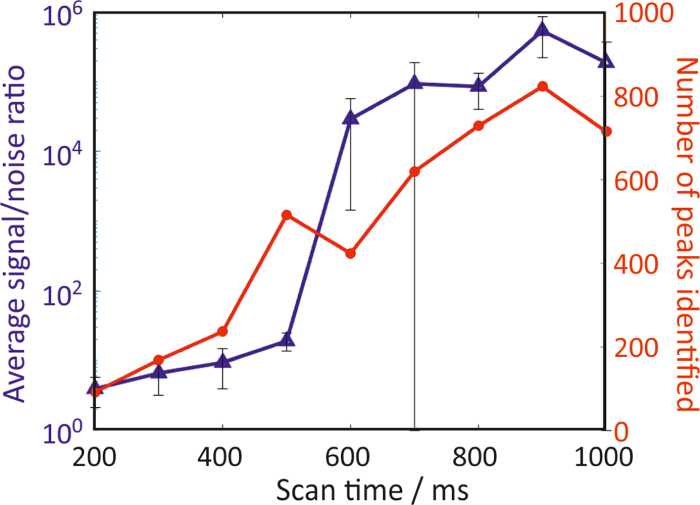
The average signal-to-noise ratio (blue) and number of peaks identified (red)for scan times in the range from 200 to 1000 ms. Data were averaged over five repeat measurements, with the exception of the 800 ms data, which were averaged over four repeats due to an anomalous measurement. One-standard-deviation error bars in the signal-to-noise are shown.

The effect of employing different scan speeds was assessed by considering both the signal-to-noise ratio and the number of m/z peaks observed, with the aim being to maximise both of these quantities. For this purpose, a ‘peak’ was identified as any signal more than three times the standard deviation of the baseline, while the signal-to-noise ratio was defined as the average ratio between the intensity of each identified peak and the standard deviation of the baseline. Fig. 4 shows that the scan time that provided the highest values for both quantities of interest was 900 ms, though the performance is similar across a range of scan times from 600–1000 ms. A scan time of 900 ms was used in subsequent measurements.

### 3.4 Scan range

As noted previously, the Advion ASAP-MS instrument has a full scan range spanning 10 to 1200 m/z. However, in our measurements on blood plasma, no significant signal was observed above 1000 *m/z*, so the scan range in subsequent measurements was reduced to 10–1000 m/z. Reducing the scan range has the result that for a given scan time, the dwell time on a given m/z is increased, increasing the number of ions detected and improving the signal-to-noise ratio. We note that the ionisation efficiency and transport of ions through the instrument, and therefore the signals detected as a function of mass, can vary significantly depending on the details of the instrument design and settings, so the appropriate mass range over which to scan should be determined separately for each new instrument and sample type.

### 3.5 Acquisition time

Having chosen a scan speed, the acquisition time, i.e. the amount of time for which the probe is inserted into the mass spectrometer, determines the number of spectra that will be recorded during each measurement. One of the goals of the present work is to develop a rapid protocol suitable for making measurements on large numbers of samples in a clinical setting, and choosing a suitably short acquisition time is therefore a key consideration. However, the acquisition time should be long enough to capture signals from both volatile and non-volatile species within the sample, and to generate enough spectra to ensure that averaging the spectra over the acquisition time yields an acceptable signal-to-noise ratio. It is also worth noting that if the acquisition time is too long then the sample can start to burn on the surface of the capillary, after which artefacts may appear in the spectra, and any further information gained will be minimal.

The optimum acquisition time was determined via an Allan variance [27], [28] analysis, as follows. To generate a suitable data set for the analysis, measurements were made on two plasma samples. For each sample, three repeat measurements were made with an acquisition time of 60 s for each repeat. For these measurements the scan range was set to 10–1200 m/z and the scan time was 500 ms. These are slightly different from the previously determined optimum settings for these two parameters. The scan range was set to the full mass range of the instrument in order to pick up any new high-mass peaks appearing as a result of sample degradation, and the scan time was reduced in order to increase the number of scans per acquisition cycle for the purposes of the Allan variance analysis.

**Fig. 5:**
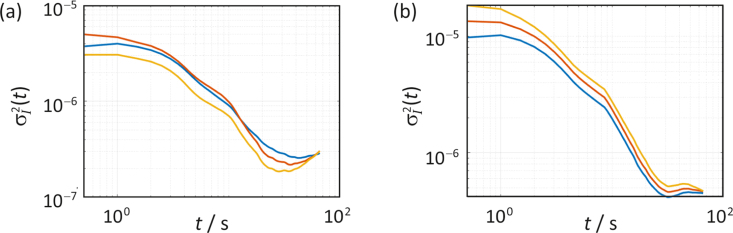
Allan variance as a function of acquisition time t for two plasma samples, (a) and (b). Three repeat measurements for each sample are shown by the blue, yellow, and red lines.

The Allan variance is used to determine the intrinsic noise in a measurement of variable x (in our case x is the m/z peak intensities) as a function of the averaging time, t.

(1) $$\sigma_{x}^{2}\left(t\right)=\frac{1}{2\left(N-1\right)}\overset{N-1}{\underset{i=1}{\sum }}{\left(x_{i+1}-x_{i}\right)}^{2}$$

where N is the total number of measurements made during the acquisition time and i is the index of a given measurement. Plotting the Allan variance as a function of the acquisition time (or number of measurements) quickly reveals the optimum acquisition time. At short times the variance is dominated by measurement-to-measurement noise, and therefore reduces rapidly as the measurement time is increased and more measurements are averaged. At long times the variance becomes dominated by experimental drift, and begins to increase again. The optimum measurement time is the time at which the curve passes through a minimum. Allan variance plots for each repeat measurement on the two samples are shown in Fig. 5. Based on the plots, the optimum acquisition time was determined to be between 20 and 30 s, and an acquisition time of 25 s was therefore chosen for all subsequent measurements.

We note that in addition to standardising the acquisition time, it is important to leave enough time in between repeat measurements for the signal to fall back to baseline levels. Failing to do this can lead to problems in automating the data processing, as well as possible contamination of spectra with residue from previous samples. Fig. 2(d) show chromatograms for a data set in which such problems arise.

### 3.6 Number of repeat measurements

Averaging the mass spectra over a number N of repeat measurements for each sample improves both the signal-to-noise and the reproducibility of the measurement. For a given sample, while we expect some variation between individual repeat measurements, we expect the spectra to converge to a constant form as N is increased. To determine the number of repeats after which this convergence is achieved, a data set was generated comprising 50 repeat measurements on a single sample. The spectra were recorded over a mass range of 10–1000 m/z, with a scan time of 500 ms and an acquisition time of 25 s. For each value of N from 2 to 50, a set of N spectra were chosen randomly from the 50 measurements, and averaged. This process was repeated 100 times to obtain a set of 100 averaged spectra for each value of N. For each N, the mean peak intensity ${\overset{¯}{I}}_{m/z}\left(N\right)$ and standard deviation across the 100 averaged spectra were determined for each m/z peak. The means were used to determine the summed fractional change $\Delta I_{N}$ in peak intensity across all mass peaks when N is incremented by one, according to the following expression:

(2) $$\Delta I_{N}=\underset{m/z}{\sum }\frac{{\overset{¯}{I}}_{m/z}\left(N\right)-{\overset{¯}{I}}_{m/z}\left(N-1\right)}{{\overset{¯}{I}}_{m/z}\left(N-1\right)}$$

**Fig. 6:**
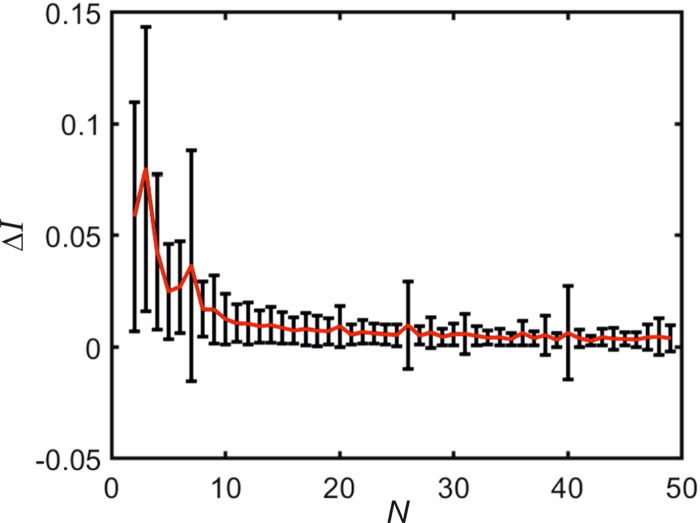
The change in average normalised intensity (ΔI) across all m/z peaks as a function of number of repeats averaged (N). One-standard-deviation error bars are shown.

The plot of Eq. (2) against N shown in Fig. 6 reveals how the averaged spectra change with the addition of further measurements. We see that the variation in signal decreases rapidly over the range from N=1 to N=10, and then remains reasonably constant for larger N. Based on this analysis, we conclude that averaging the data over 10 repeat measurements for each sample provides a good balance between optimising the reproducibility and quality of the measurement and maintaining a rapid measurement protocol. As noted earlier, after five repeat uses of the same capillary, residue on the capillary tip begins to affect the background measurement. Consequently, the ten repeat measurements were obtained as two cycles of five repeat measurements, with a change of capillary in between the two cycles.

The complete optimised measurement protocol can be found in Appendix.

## 4 Example data obtained using the optimised protocol

**Fig. 7:**
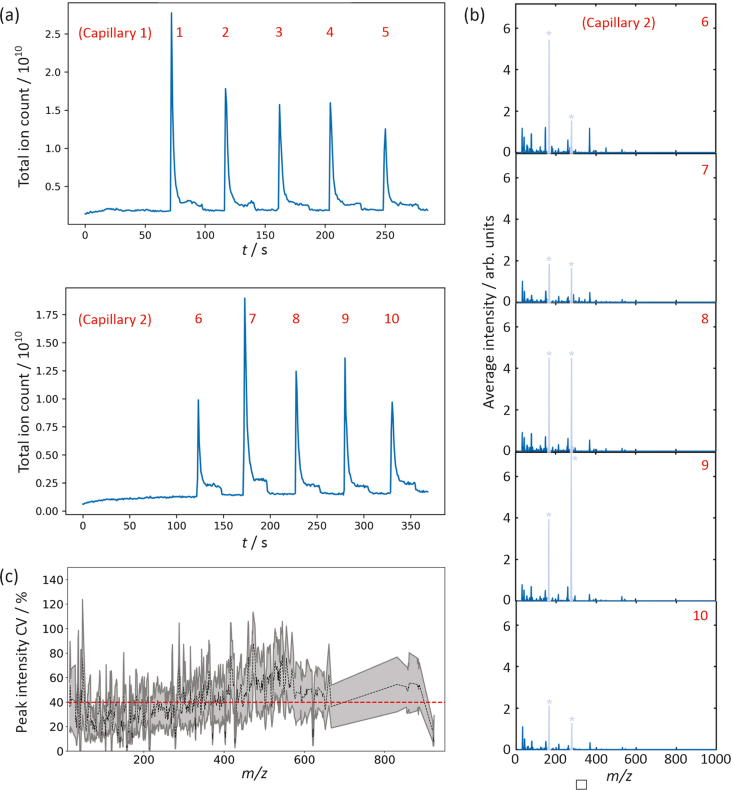
(a) Chromatograms for a set of ten repeat measurements on an OxAAA sample recorded using the optimised method. The measurement number for each replicate measurement is labelled in red above the relevant peak in the chromatogram. The tip of the ASAP probe was replaced with a clean glass capillary after measurement 5, i.e. measurements 1–5 and 6–10 employed two different tips; (b) Examples of individual mass spectra (in this case for measurements 6–10 recorded with the second capillary). Known contaminant peaks are greyed and marked with an asterisk; (c) Mean (black dashed line) and standard deviation (grey solid region) of the coefficient of variance in m/z peak intensities, determined as described in the text. The average coefficient of variance across the entire spectrum is 39.9%, shown as a red dashed line on the plot.

A sample data set recorded for a plasma sample using the optimised measurement protocol is shown in Fig. 7. Chromatograms for all measurements are included, with example mass spectra (for the second set of five measurements) shown in order to provide a qualitative indication of the reproducibility of individual measurements.

Considering first the chromatograms, we see that during the background measurement at the start of each trace, the intensity is low and stable. The ten repeat measurements on the sample yield well defined spikes in signal, with an initial sharp rise and a slower decay.^1^ The peak height observed in each repeat is primarily a function of the precise amount of plasma transferred to the capillary tip of the ASAP probe, and since this step is performed manually this varies from measurement to measurement. The initial sharp rise observed on each insertion of the ASAP probe into the instrument is largely associated with desorption of cholesterol. Intact cholesterol has m/z386.35, and we observe protonated cholesterol in some of our spectra at m/z387.4. However, one of the most intense and variable peaks in the mass spectrum is the dehydrated cholesterol peak at m/z369 resulting from water loss from the molecular ion. In between each measurement, and after the final measurement, the total ion count returns rapidly to the baseline intensity level. This indicates that sample ions have cleared the spectrometer and that the instrument is clean. The clear, well-separated signals in the chromatogram make it very straightforward to automate the extraction of individual measurements from the data file.

Next we consider the examples of individual mass spectra shown in Fig. 7(b), each of which are averaged over the 25 s measurement time during which the ASAP probe was present in the ion source. We see that though there are two intense peaks whose intensity varies considerably (marked with asterisks and plotted in a lighter colour to guide the eye), overall the mass spectra are reasonably reproducible from measurement to measurement. We believe that the two asterisked peaks are contaminant peaks arising from alkyl phthalate plasticisers, most probably originating from the nitrogen line used to supply N_2_ from the laboratory nitrogen source to the ion source of the mass spectrometer [29].

To gain a more quantitative insight into the degree of reproducibility, ten repeat measurements were made on each of 20 plasma samples donated by healthy volunteers as part of the OxAAA study. For each measurement, the time-resolved mass spectra were averaged over the 25 s acquisition time to give a single mass spectrum, yielding 10 time-averaged mass spectra for each of the 20 samples. These 10 mass spectra were used to calculate the coefficient of variation (CV) for each sample as a function of m/z, defined as the ratio of the standard deviation to the mean across the 10 repeat measurements for each m/z value in the mass spectrum with an intensity greater than a defined minimum intensity threshold of $5\times 10^{5}$ counts. The mean and standard deviation of the resulting CV(m/z) functions over the 20 different samples then provide a reasonable measure of the typical variability in the measurements. Fig. 7(c) shows the result of this analysis.

Inspecting the plot of the averaged coefficient of variation, we see that CV is fairly constant across all peaks, perhaps increasing somewhat with m/z. In line with other studies [30], we find that the CV varies considerably with m/z, ranging from 20%–50%, with an average value of 39.9%. For comparison, coefficients of variation found in much more sophisticated LC-MS metabolomics studies on human plasma are typically in the range from 10 to 30% [31], with CVs in the range 20 to 30% generally deemed ‘acceptable’.

There is scope to improve our CV further. For example, we note that when we have just cleaned the Advion instrument, the measured CV increases over the first few measurements, fairly quickly settling at the value of around 40% noted above. If the ion source is in need of cleaning then the CV rises to much higher values. In a small number of measurements we were able to make with an alternative instrument (Waters Radian ASAP) on short-term loan, we were able to achieve a mass-averaged CV of 25%. Taken together, these results imply that details of the ion source design may have quite a significant impact on the achievable CV, and that it would be well worth investing some effort into performing a rigorous study into the relative performance of different instruments in order to identify which ion source features are most important in this regard. Identifying and eliminating (or masking) background peaks as far as possible may provide another route to improving the overall CV, since these peaks arise from a variety of sources and are often quite variable in intensity. Finally, we could improve our CV significantly simply by thresholding the mass spectra, since we have many low-intensity peaks that are quite variable in intensity. However, we have found in other studies [32] that some of these peaks are very important in characterising our plasma samples despite their variability, so this approach would come at the expense of losing potentially valuable information.

## 5 Conclusions

An optimised method has been developed for the measurement of human blood plasma using ASAP-MS. This method will enable untargeted metabolomics profiles to be recorded for patients with a range of diseases. The optimised protocol allows rapid measurements to be made by a variety of operators, and does not require extensive prior experience or training in mass spectrometry. The coefficient of variation between measurements is somewhat higher but not too far out of line with the range commonly observed when using other techniques to analyse human plasma, and there is scope to reduce this further. Based on our experience to date, we believe that ASAP-MS is a promising technique for translation into clincal settings, and has considerable potential to be used in clinical diagnostic and prognostic assessments.

### Consent to participate

Informed consent was obtained from all individual participants included in the study.

### Ethics approval

All procedures performed in studies involving human participants were in accordance with the ethical standards of the institutional and national research committee and with the 1964 Helsinki declaration and its later amendments or comparable ethical standards.

### Consent for publication

Not applicable.

### Code availability

The software used to process the data is available on request via the corresponding author.

### Funding

University of Oxford John Fell fund, Wellcome Trust grant code 218514/Z/19/Z, Academy of Medical Sciences Starter Grant SGL013/1015, UKRI Future Leaders Fellowship MR/V025775/1.

## CRediT authorship contribution statement

**Annabel S.J. Eardley-Brunt:** Writing – review & editing, Writing – original draft, Validation, Software, Methodology, Investigation, Formal analysis, Data curation, Conceptualization. **Anna Jones:** Investigation, Formal analysis, Conceptualization. **Thomas Mills:** Investigation, Formal analysis, Conceptualization. **Liwen Song:** Software, Methodology, Investigation, Formal analysis, Conceptualization. **Rafail Kotronias:** Resources, Investigation, Data curation. **Pierfrancesco Lapolla:** Resources, Data curation. **Ashok Handa:** Resources, Data curation. **Regent Lee:** Writing – review & editing, Resources, Data curation, Conceptualization. **Keith Channon:** Resources, Methodology, Funding acquisition, Data curation. **Giovanni Luigi de Maria:** Resources, Methodology, Data curation, Conceptualization. **Claire Vallance:** Writing – review & editing, Writing – original draft, Supervision, Resources, Project administration, Methodology, Investigation, Funding acquisition, Conceptualization.

## Declaration of competing interest

The authors declare that they have no known competing financial interests or personal relationships that could have appeared to influence the work reported in this paper.

## Data Availability

Data will be made available on request.

## Appendix Optimised measurement protocol for ASAP-MS measurements on human plasma

The ASAP-MS instrument is set up in positive ion mode with the ion source settings set to ‘high temperature, low fragmentation’ mode. The mass range is selected to be 10–1000 m/z, and the scan time is set to 900 ms. The acquisition run is started.

A clean capillary is mounted into the tip of the ASAP instrument probe. The probe is inserted into the probe slot in the ion source housing for 30 s, and a background or blank spectrum is recorded for the clean capillary during this time. The background spectrum is subtracted from each sample measurement during the data processing. The probe is then removed from the spectrometer, cleaned and cooled with methanol, and dried on a lens tissue.

The plasma sample to be measured is thawed and vortex mixed. The probe is then used to wipe the inside surface of the sample container approximately 5 mm above the sample surface. The probe is then reinserted into the mass spectrometer for a period of 25 s, during which a series of mass spectra are recorded for the sample. The probe is then removed from the mass spectrometer and cleaned with ethanol and lens tissue ready for the next repeat measurement. Five repeat measurements are made with the same capillary. After this the complete set of five measurements, including background measurement, is repeated with a new clean capillary, for a total of ten measurements per sample.

## Footnotes

1 For the benefit of others developing similar protocols for different samples, we note that not all biological samples give rise to the same peak shape in the ion chromatogram. In particular, samples with a high fat content tend to yield more variable peak shapes.
